# Sputnik V-Induced Antibodies against SARS-CoV-2 Variants during the Dissemination of the Gamma Variant in Venezuela

**DOI:** 10.3390/v16091480

**Published:** 2024-09-18

**Authors:** Christopher Franco, Alejandro Cornejo, Mariajosé Rodríguez, Alexis García, Inirida Belisario, Soriuska Mayora, Domingo José Garzaro, Rossana Celeste Jaspe, Mariana Hidalgo, Nereida Parra, Ferdinando Liprandi, José Luis Zambrano, Héctor Rafael Rangel, Flor Helene Pujol

**Affiliations:** 1 Laboratorio de Virología Celular, Centro de Microbiología y Biología Celular, Instituto Venezolano de Investigaciones Científicas (IVIC), Caracas 1020, Venezuela; chrfranco.94@gmail.com; 2 Laboratorio de Bioquímica Celular, Centro de Microbiología y Biología Celular, Instituto Venezolano de Investigaciones Científicas (IVIC), Caracas 1020, Venezuela; cornejo.alejandro@gmail.com; 3 Laboratorio de Virología Molecular, Centro de Microbiología y Biología Celular, Instituto Venezolano de Investigaciones Científicas (IVIC), Caracas 1020, Venezuela; rodriguez95mariajose@gmail.com (M.R.); dgarzaro@gmail.com (D.J.G.); rossanajaspesec@gmail.com (R.C.J.); hrangel2006@gmail.com (H.R.R.); 4 Instituto de Inmunología, Universidad Central de Venezuela (UCV), Caracas 1020, Venezuela; alexisgarcia27@gmail.com (A.G.); ibelisariogomez@gmail.com (I.B.); sori_mayo@hotmail.com (S.M.); 5 Laboratorio de Inmunoparasitología, Centro de Microbiología y Biología Celular, Instituto Venezolano de Investigaciones Científicas (IVIC), Caracas 1020, Venezuela; mariana.hidalgo.r@gmail.com; 6 Laboratorio de Fisiología de Parásitos, Centro Biofísica y Bioquímica, Instituto Venezolano de Investigaciones Científicas (IVIC), Caracas 1020, Venezuela; bionereida@gmail.com; 7 Laboratorio de Biología de Virus, Centro de Microbiología y Biología Celular, Instituto Venezolano de Investigaciones Científicas (IVIC), Caracas 1020, Venezuela; fliprand@gmail.com

**Keywords:** SARS-CoV-2, Sputnik V, antibodies, variants, ELISA, neutralization

## Abstract

The COVID-19 pandemic was characterized by the emergence and succession of SARS-CoV-2 variants able to evade the antibody response induced by natural infection and vaccination. To evaluate the IgG reactivity and neutralizing capacity of the serum of individuals vaccinated with Sputnik V (105 volunteers vaccinated) against different viral variants. IgG reactivity to the Spike protein (S) was evaluated by ELISA. A plaque reduction neutralization test was performed using different viral variant isolates. At 42 days post-vaccination, the frequency of recognition and reactivity to the S protein of the Omicron variant was lower compared to that of the other variants. In general, a higher average neutralization titer was seen against the ancestral variant compared to the variants, especially Omicron. However, some sera exhibited a higher neutralization titer to the Gamma variant compared to the ancestral variant, suggesting unapparent exposure during the clinical trial. Antibodies induced by Sputnik V can recognize, persist, and neutralize SARS-CoV-2 variants, with Omicron being the one that best evades this response. These results represent a unique report on the humoral response induced by a globally lesser-studied vaccine in terms of efficacy and immune escape, offering insights into developing vaccines targeting unknown coronaviruses.

## 1. Introduction

By March 2024, SARS-CoV-2, the virus responsible for the COVID-19 pandemic, had caused more than 700 million cases and claimed over 7 million lives [1], despite estimates suggesting even higher figures [2,3]. Notably, this viral family possesses a unique non-structural protein with proof-reading capacity, setting it apart from other RNA viruses. The virus has exhibited many mutations due to numerous replication cycles in millions of hosts, a high frequency of recombination, and the effect of host editing enzymes [4].

Consequently, variants with a higher transmission capacity and immune evasion have been selected [5]. Five variants of concern (VOCs) of SARS-CoV-2 have emerged since the end of 2020, resulting in new waves of infections, deaths, and chronic sequelae [6]: Alpha (original lineage B.1.1.7), which arose in the UK; Beta (B.1.351), which appeared in South Africa; Gamma (B.1.1.28.2, P.1), which originated in Brazil; Delta (B.1.617.2), from India; and Omicron (B.1.1.529), first identified in South Africa and whose sub-lineages are the only ones circulating at present [7]. Furthermore, antigenic evolution of the S protein has dampened the efficacy and effectiveness of many vaccines [8].

Non-replicating adenoviral vectors, mRNA, inactivated whole viruses, and protein subunit-based vaccines are powerful tools in the fight against emergent pathogens, including SARS-CoV-2. Prior to the introduction of bivalent adapted boosters, most vaccine-approved candidates targeted the S protein of the ancestral variant that was first identified in China [9]. The Gam-COVID-Vac (Sputnik V) vaccine has played a significant role in vaccination campaigns in Venezuela and other Latin American countries. Studies have consistently shown that it is as effective as mRNA vaccines [10]. This vaccine represented 23% of the given doses in Venezuela (approximately 8.7 million out of almost 38 million, including first and second doses) [11]. Sputnik V is composed of two non-replicative recombinant human adenoviruses (serotypes 26 and 5), both of which carry a coding sequence for the ancestral S protein. The vectors have deletions in the early expression genes E1 and E3, which prevent their replication and permit the introduction of the gene coding for the SARS-CoV-2 S glycoprotein. Additionally, the heterologous design of Sputnik V is advantageous, as it reduces immunogenicity issues arising from immunity against the vector [12]. Upon delivery, each antigenic stimulus produces antibodies through plasma cells, diversifies the B cell receptor (BCR) repertoire, and generates specific B memory cells against the entire S glycoprotein [13,14,15].

Understanding how the recognition and neutralization of antibodies generated after Sputnik V vaccination may be affected by variants of this virus is an issue that requires further research. In contrast to mRNA-based vaccines, the effects of the Sputnik V vaccine have been significantly less studied. In fact, a PubMed query shows a scarce number of publications about the Sputnik V vaccine compared to the other most common ones, even though its use was approved in more than 70 countries, most of them developing countries (Table 1). In Venezuela, a clinical trial was conducted using the Sputnik V vaccine from December 2020 to June 2021, a time that coincided with the emergence of the Gamma VOC in the country [16]. The aim of this study was to evaluate the antibody reactivity and neutralization ability of SARS-CoV-2 variants and to assess the correlation between these parameters in vaccines in Venezuela.

## 2. Materials and Methods

### 2.1. Experimental Design, Population and Sample 

Sera samples from the prospective, double-blind, randomized, placebo-controlled clinical trial of Sputnik V in Venezuela from December 2020 to July 2021 (NCT04642339) were evaluated. The inclusion and exclusion criteria are detailed on the clinical trial website (clinicaltrials.gov/ct2/show/NCT04642339). Volunteers were residents of Caracas, aged 20–80 years, and all signed an informed consent form approved by the National Ethics Committee for Research on COVID-19. Samples were collected 42 days after vaccination (dpv), counting after the first dose and 21 days after the second dose. Matching samples from 46 vaccinees were also collected at 180 dpv. Of the 133 volunteers, 105 were vaccinated and 28 received a placebo. Sera from 85/105 vaccinees without serological evidence of exposure, as determined by nucleoprotein (N) reactivity [19], were analyzed for reactivity to the S and RBD antigens at 42 dpv. For comparisons of 42 versus 180 dpv reactivity, 20 serum samples from N-negative vaccinees were selected. Eighteen samples from this subgroup were used for neutralization assays at 42 and 180 dpv.

### 2.2. Antigens

The recombinant antigens for the enzyme-linked immunosorbent assays (ELISA) were acquired from MyBioSource Inc. (San Diego, CA, USA). The ancestral S protein (MBS8574721) and RBD (MBS8574741), Alpha S (MBS184025), Beta S (MBS184021), Gamma S (MBS184022), RBD (MBS434292), Delta S (MBS184024), and Omicron S (MBS553745) antigens were employed. The concentration of antigens was determined using the Qubit™ Protein Assay according to the manufacturer’s specifications (catalog number Q32866, Thermo Fisher, Waltham, MA, USA).

### 2.3. Reactivity toward SARS-CoV-2 Antigens

To assess the differences between reactivity against S or RBD from SARS-CoV-2 variants, an adapted version of the protocol proposed by Stadlbauer et al., 2020 was used [20] and thoroughly described by Cornejo et al., 2024 [19]. Briefly, 96-well microtiter plates were coated with 1 µg/mL of each capture antigen. To prevent non-specific antibody binding, a commercial blocking solution was employed (Abcam, Cambridge, UK, ab126587). All samples were diluted 1/100 and incubated for 2 h at 37 °C. Horseradish peroxidase (HRP) IgG-linked (Jackson ImmunoResearch Inc., West Grove, PA, USA) was used as a detection antibody (1/70,000) and incubated for 1 h. TMB (3,3′,5,5′-tetramethylbenzidine) was used as the chromogenic substrate for HRP. All washes were performed with 0.01% PBS-Tween. HCl [3 M] was used to stop the reaction. Data collection was performed using a spectrophotometer (SpectraMax 250, Hampton, NA, USA) at 450 nm. Reactivity against SARS-CoV-2 antigens was assessed within this group of 134 individuals. The optical densities (O.D.) of the blanks were subtracted from those of the O.D. of the samples. O.D. exceeding the cut-off established with the negative control mean plus 3 standard deviations were considered as responders to each antigen. A negative control was established using 18 prepandemic sera from apparently healthy individuals. A mixture of two serum samples from individuals with hybrid immunity (Sputnik V-vaccinated and two confirmed infections by PCR) was used as a positive control. Both controls were used during normalization to estimate the relative IgG levels using the sample-to-positive ratio (S/P), as previously described [21]:

$$\frac{S}{P}=\frac{\left(O.D.\;sample-O.D.\;negative\;control\right)}{\left(O.D.\;positive\;control-O.D.\;negative\;control\right)}\times 100$$

### 2.4. Plaque Reduction Neutralization Test of Sputnik V Vaccinees Sera

A plaque reduction neutralization test (PRNT) was conducted on 18 samples of serum collected at 42 and 180 dpv. PRNT was performed according to a previously reported procedure [19,22]. VERO C1008 cells (Vero 76, clone E6, vero. ATCC, Manassas, VA, USA) were maintained at 37 °C and 5% CO_2_ in RPMI medium (Thermo Fisher Scientific, 11875093) supplemented with 10% fetal bovine serum (FBS, Thermo Fisher Scientific, 16000044) and 1% penicillin–streptomycin (Thermo Fisher Scientific, Waltham, MA, USA). All procedures were performed in a biosafety cabinet class II (ESCO^®^, Airstream^®^, Singapore) within a level 3 biosecurity laboratory. Viral seeds of the different SARS-CoV-2 variant strains, ancestral (B.1.1.33), P.1 (Gamma VOC), AY122 (Delta VOC), B.1.621 (Mu VOI), and BA.1.1 (Omicron VOC) [18], were incubated for 30 min at 37 °C with different dilutions of vaccinated and control sera. The infection process was performed for one hour at 37 °C in a 5% CO_2_ atmosphere, and the cells were washed twice with PBS to remove non-internalized viruses. The cells were overlaid with 0.5% carboxymethyl cellulose in culture medium and incubated for 72 h. Then, the cells were fixed with 4% formaldehyde and stained with crystal violet to count the number of lytic plaques under each condition. A highly responsive mix of hybrid sera was used as the positive control, whereas culture medium without serum or viral seeds was used as the mock control. The PRNT50 of each serum sample was determined through a nonlinear regression test, defining this value as the reverse of the dilution at which 50% of the virus is neutralized [19].

### 2.5. Statistical Analysis

To evaluate differences between reactivities against the different antigens at 42 dpv, the Kruskal−Wallis test (*p* < 0.05) was used with Dunn’s post-hoc analysis. For comparisons between 42 and 180 dpv, a Wilcoxon matched-pair signed rank test was carried out to estimate significant differences in reactivity between both times (*p* < 0.05). To establish differences among neutralization titers toward the different SARS-CoV-2 isolates within 42 and 180 dpv, a Wilcoxon matched-pairs signed rank test was conducted (*p* < 0.05, PRISM GraphPad 9.0 ©, La Jolla, CA, USA). Statistical difference was assessed using the Chi-square test (Epi Info™, Centers for Disease Control and Prevention, Atlanta, GA, USA) for frequency comparisons among groups. Association between vaccinees ages, IgG antibody reactivity, and PRNT50 was determined through a hierarchical clustering correlation matrix heatmap by the Spearman method with a confidence interval (CI) of 95% employing the “visdat” and “ggcorrplot” packages of R version 4.3.3. To explore the patterns of antibody response, an ascending hierarchical classification of the individuals was performed using the “shiny” package in R version 4.3.3.

## 3. Results

### 3.1. Reactivity to SARS-CoV-2 Variants S Protein

**Figure 1:**
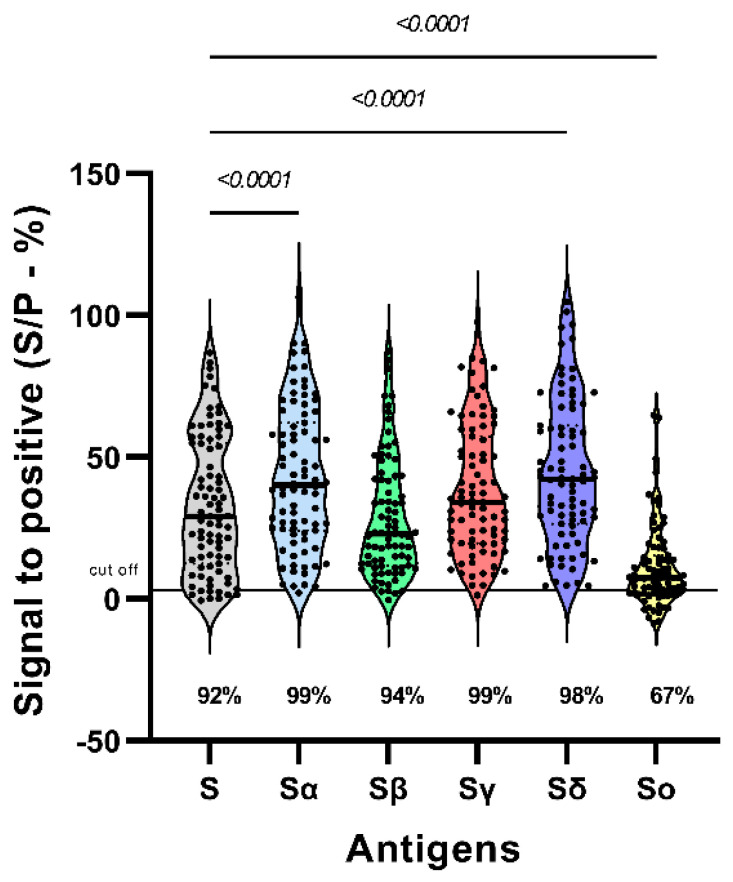
Reactivity of SARS-CoV-2 variants to the S protein after 42 dpv. Differences between the reactivities of IgG antibodies from Sputnik V vaccinated individuals toward S (ancestral), Sα (alpha), Sβ (beta), Sγ (Gamma), Sδ (Delta) and Sο (Omicron) were determined by indirect ELISA (n = 85). The percentage of responders is shown at the bottom of the violin plots. Significant differences were assessed using Kruskal-Wallis and Dunn’s post-hoc test. The *y*-axis represents the ratio of each sample’s signal to the signal of the positive control, while the *x*-axis represents each SARS-CoV-2 variant.

The reactivity to S from the five VOCs was assessed by ELISA in 85 vaccinated volunteers without evidence of exposure to the virus. Statistically significant differences were observed between the mean reactivities of the S protein of the different variants. Reactivity to the S protein of the Alpha and Delta variants was higher than that observed for the ancestral variant (*p* < 00001). No significant differences were observed between the reactivity to the S protein of the ancestral variant and that of the Beta and Gamma variants. The mean reactivity to the ancestral variant was higher (3-fold) than that to the Omicron variant (*p* < 00001). Almost all sera reacted to the S protein of the different variants, except for the Omicron variant, which was only recognized by 67% of the sera at a statistically lower frequency (*p* < 0.0001) (Figure 1).

**Figure 2:**
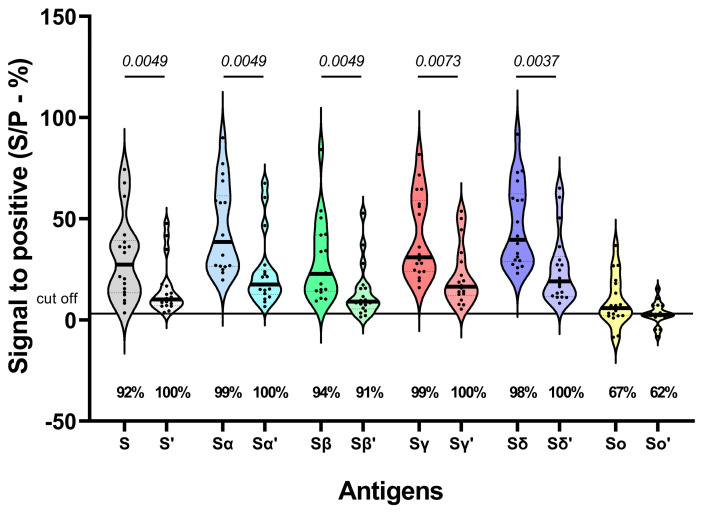
Reactivity of SARS-CoV-2 variants to the S protein after 42 and 180 dpv (n = 18). The points on the graph represent the same set of samples tested for each antigen. Antigens with a prime symbol correspond to 180 dpv. The percentage of respondents is shown below each violin plot. Wilcoxon test was performed to estimate significant differences in reactivity between both times.

The reactivity to S VOCs was also assessed in follow-up sera. The S/P values were lower at 180 dpv, with a reduction of 3-fold (S ancestral), 2-fold (S Alpha, Beta, Gamma, and Delta), and 3-fold (S Omicron). However, no reduction was observed in the number of sera recognizing each variant; an even higher frequency of sera recognizing the S protein was observed. The exception was the Omicron variant, for which even fewer sera recognized the protein, although this difference was not significant (Figure 2).

### 3.2. Neutralizing Activity of Sputnik V-Induced Antibodies against SARS-CoV-2 Variants at 42 and 180 dpv

**Figure 3:**
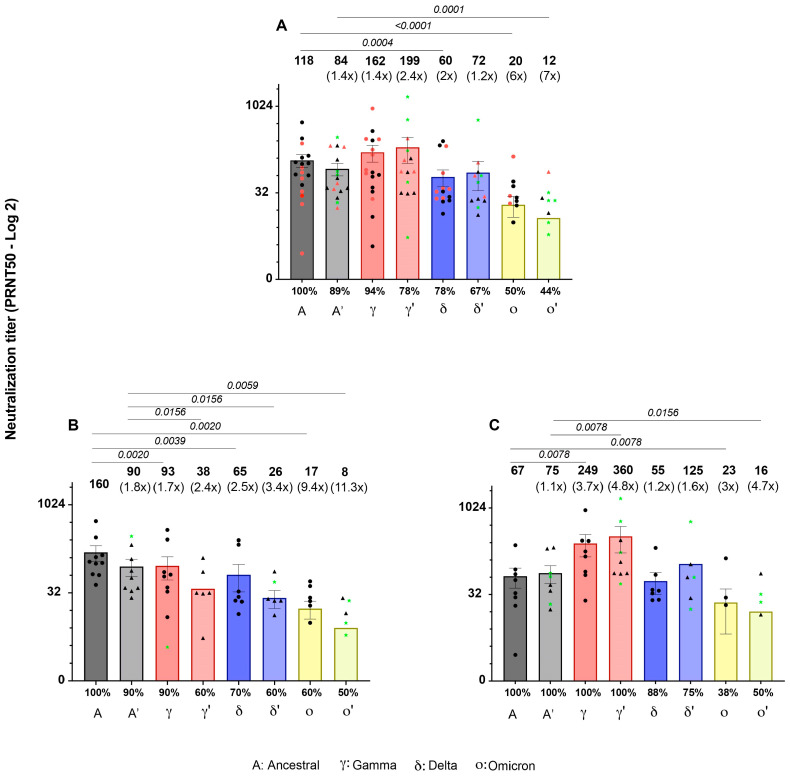
Neutralization titers of sera from Sputnik V vaccinees 42 and 180 dpv against SARS-CoV-2 variants. (**A**) Sera with higher titers against Gamma VOC with respect to the ancestral variant are highlighted in red, while those that did not show this behavior are highlighted in black (n = 18). (**B**) Sera with higher titers against the ancestral variant than against the other variants (n = 10). (**C**) Samples that elicited a higher titer against Gamma compared to the ancestral variant (n = 8). Each symbol represents an individual sample and indicates the titer required to neutralize 50% of the lytic plaques, together with the standard error of the mean. Green stars indicate samples whose PRNT50 after 180 dpv was higher than that after 42 dpv. The numbers in brackets indicate the factor of increase or decrease in the neutralization titer against each variant compared to the ancestral variant. The percentage of responders is shown at the bottom of each bar. Greek letters with a prime symbol correspond to 180 dpv. Wilcoxon test was performed to estimate significant differences in PRNT50 between variants and times. Although some points equal to 0 are not plotted, their values are included in the analysis.

The plaque reduction neutralization titer was assessed on 18 serum samples taken at 42 and 180 dpv from vaccinees with no signs of infection by the virus (Figure 3A). A modest reduction in the neutralization titer was observed with the Delta variants at 42 and 180 dpv compared to the ancestral variant, while the mean titer of neutralization against the Gamma variant was higher compared to the ancestral variant, although this difference was not significant. In the case of Omicron, a significant reduction in the neutralization titer was observed at 42 and 180 dpv, by 6 and 7 times, respectively, in addition to a significant reduction in the number of sera neutralizing the variant.

When dissecting the neutralizing activity against the Gamma variant, a group of sera (n = 10) exhibited a modest decrease in neutralizing titers against it at 42 and 180 dpv (Figure 3B), while a 3.7-fold increase in neutralization titer was observed against this variant in the sera of the other group (n = 8) at 42 and 180 dpv compared to the ancestral variant (Figure 3C).

**Figure 4:**
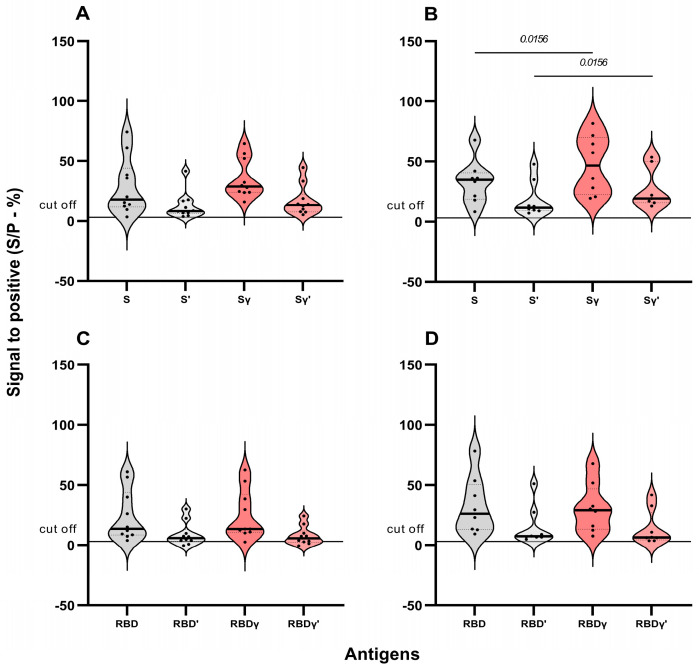
Serology of low and high neutralizers of the Gamma variant 42 vs. 180 dpv. Differences between antibody reactivities are shown. (**A**) Reactivities against S protein of sera with a higher neutralization titer to the ancestral variant compared to Gamma (n = 10). (**B**) Reactivities toward S protein of sera with higher titers against Gamma are shown (n = 8). (**C**) Reactivities against RBD of higher neutralizers to ancestral variant compared to Gamma (n = 10), and (**D**) higher neutralizers to Gamma variant compared to ancestral (n = 8). Antigens with a prime symbol correspond to 180 dpv. The Wilcoxon test was performed only to point out comparisons between ancestral and Gamma reactivities.

Because of the unexpected higher neutralization activity in some sera of the Gamma variant with respect to the ancestral strain, the reactivity to the S and RBD antigens of the SARS-CoV-2 variants was evaluated for the sera analyzed for neutralizing activity. A statistically significant higher reactivity to the S Gamma variant, compared to the ancestral protein, was observed in the sera exhibiting higher neutralizing activity against this variant (Figure 4A,C). This difference was not observed in the reactivity to RBD (Figure 4B,D).

### 3.3. Correlation between Age, Reactivity and PRNT50

**Figure 5:**
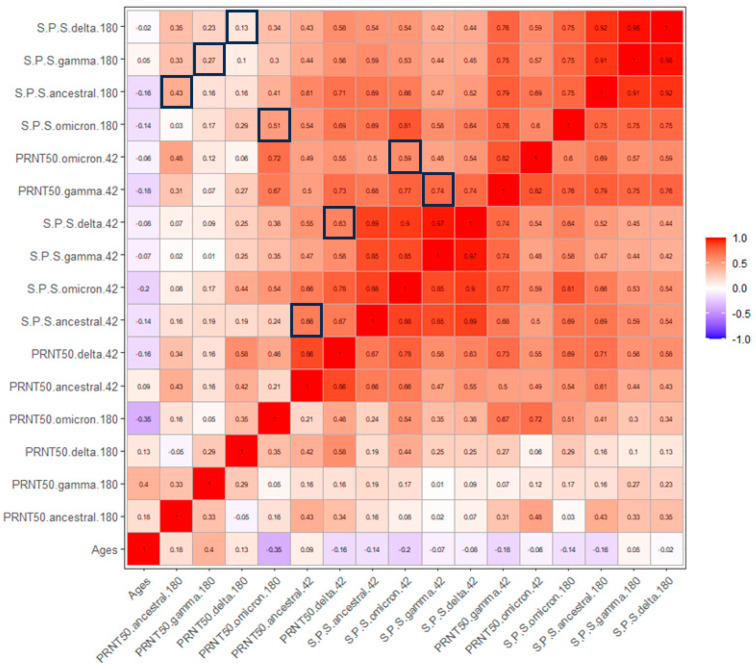
Correlation matrix between ELISA reactivity, plaque reduction neutralization titer, and age at 42 and 180 dpv. The Spearman correlation coefficient is shown in white (>0.6) and black (<0.6). The degree of association between these variables is represented by a heatmap, where blue and red represent positive and negative correlations, respectively. All correlations are statistically significant, except for correlations with subject age. Black squares indicate correlations between reactivities and PRNT50 (n = 18).

The Spearman test was performed to correlate all the parameters analyzed in this study. There was no significant correlation between antibody levels or neutralization titers and the age of the vaccines. Regarding the associations between ELISA reactivity and PRNT50, strong (S Gamma vs. PRNT50 Gamma) to moderate positive correlations were observed at 42 dpv. Conversely, moderate (S Omicron vs. PRNT50 Omicron) to low correlations were observed at 180 dpv (Figure 5).

### 3.4. Signatures of Antibody Immune Response in Sputnik V Vaccinees

**Figure 6:**
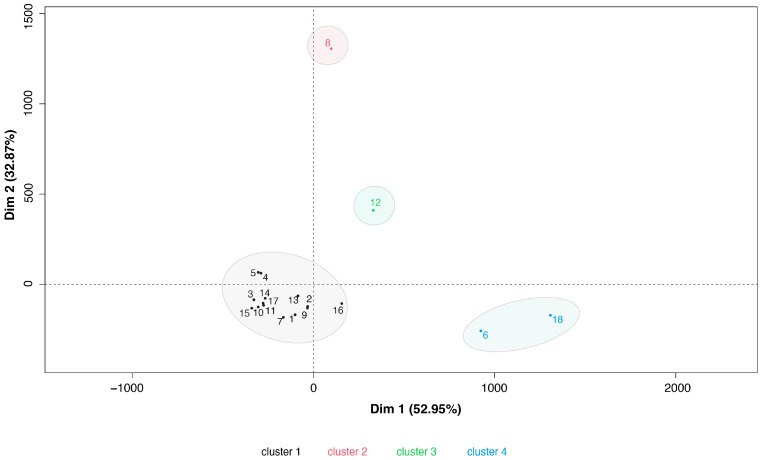
Ascending Hierarchical Classification of the individuals. Two components, denoting the percentage of variance, explained Dim 1 (52.95%) and Dim 2 (32.87%). Dim 1 and 2 represent the first two components of all reactivities and PRNT50s evaluated. They help to explain the variance observed in the system and allow us to make a classification of the samples based on their antibody response. Cluster 1 consists of low responders (n = 14). Sample 8: high responder for PRNT50 Gamma 180 dpv and PRNT50 Delta 180 dpv (descending order). Sample 12: high responder for PRNT50 Omicron 42 dpv, S/P S Beta 42 dpv, S/P S ancestral 42 dpv, S/P S Omicron 42 dpv, and S/P S Delta 42 dpv (descending order). Samples 6 and 18: high responders for S/P S Omicron 180 dpv, PRNT50 ancestral 42 dpv, PRNT50 Delta 42 dpv, PRNT50 Gamma 42 dpv, S/P S ancestral 180 dpv, and S/P S Beta 180 dpv (descending order).

An ascending hierarchical classification of vaccinees was performed to compare their patterns of reactivity and neutralization. The classification of individuals revealed two main patterns of response. One main cluster grouped most of the vaccinees (low responders, n = 14) and the remaining (high responders, n = 4) with a high reactivity and PRNT50 titer to Gamma, Delta, or Omicron variants (Figure 6).

## 4. Discussion

As vaccines are based on ancestral antigens and because of the evolution of SARS-CoV-2, it was critical to determine whether the binding, neutralization, and lifespan of antibodies induced after vaccination were affected by the new variants. Sputnik V played a pivotal role in vaccination initiatives throughout Venezuela and Latin America, so it was essential to assess the antibody response elicited by it in the face of the sequential emergence of variants of concern, considering the lesser amount of information available for this vaccine compared to mRNA ones. In order to perform comparisons of reactivity among antigens and to assess how these changed over time, we used an in-house ELISA. On the other hand, we used the PRNT to estimate the neutralization titers of these sera. We also evaluated the possible correlations between these variables.

No differences in reactivity to Beta and Gamma compared to ancestral antigens were reported. Other authors have described reduced reactivity and neutralization of Sputnik V-induced antibodies against these variants [14,23]. Interestingly, the S antigen from the Alpha and Delta variants was recognized with a higher reactivity compared to the ancestral S protein at 42 dpv, and for Delta at 180 dpv, consistent with the presence of high affinity and neutralizing antibodies generated by this vaccine against SARS-CoV-2 variant antigens [24]. With the exception of Omicron, where a significant decrease in reactivity to S and RBD antigens (RBD, was also observed at both time points, seroconversion to all other antigens ranged from 88 to 99% and correlated with the efficacy of this vaccine, as previously reported [25,26]. Although reactivity tended to decline over time, the vaccine maintained an effective response at six months. The durable antibody response induced by this vaccine has also been described by others [19,27,28]. These observations support the notion that the antibody response generated by immunization with Sputnik V persists for at least 6 months after administration of the first vaccine component. The dynamic of antibody response after Sputnik vaccination seems similar to that observed with the Pfizer vaccine [29,30].

Higher neutralization titers were reported against the ancestral variant compared to the other variants, and Omicron was the variant for which the highest reduction in neutralization titer was observed. Omicron and its sub-lineages have exhibited greater antigenic variation than previous variants, significantly altering the patterns of recognition and neutralization of vaccine-induced antibodies [31]. This change in viral behavior threatens the potential outbreaks of other emerging SARS-CoV-2 variants or new coronaviruses. Although the neutralization titer against Delta and Omicron was reduced, we observed that some sera were able to recognize and neutralize these variants. A possible explanation is that after Sputnik V injection, naïve B cells undergo germinal center (GC) selection by somatic hypermutation (SHM) of variable heavy (VH) and variable light (VL) chain genes, thus stimulating B cell receptor (BCR) repertoire diversification, increasing binding affinity, and neutralizing activity, which are considered indicators of antibody affinity maturation [32]. Indeed, mRNA vaccines induce robust B cell, germinal center, and follicular helper T cell (Tfh) responses, which increase after the second dose [33]. Furthermore, Tfhs have been detected in blood and lymph nodes for at least 200 days after vaccination [34]. In addition, memory B cell clones that interact with the RBD of the alpha, beta, and Delta variants have higher levels of SHM than those that bind only to the RBD of the ancestral variant after mRNA vaccination [35]. In addition, the persistence of GC activity after immunization, and therefore SHM, appears to promote a protective antibody response against variants of this virus [36] by increasing the likelihood of cross-neutralization of other variants, a response already reported during the convalescent phase [37,38,39].

This persistent GC activity could be explained by the antigenic persistence of adenovirus vectors after immunization in different tissues [40] and the maintenance of the S and S1 antigens, as reported in some mRNA-vaccinated individuals for up to 60 days after immunization [41]. Indeed, there is evidence that antigens are preserved in B cell follicles due to low protease activity in dendritic cells, which may explain the long-term availability of antigens after immunization [42]. Furthermore, the presence of B cells in germinal centers that recognize protein S for at least 30 weeks after immunization with mRNA has been described previously [36]. GC activity has been reported for other vaccines using both adenoviruses in Sputnik V [43,44,45]. Furthermore, an increase in memory, antibody-secreting, and Tfh cells has been reported after immunization with BNT162b2, AZD1222, and Sputnik V [46]. In addition, an increase in cytokines that mediate B and Tfh cell proliferation, differentiation, localization, interaction, and antibody class switching has been identified after Sputnik V immunization [47]. Prolonged activation of GC due to capture and prolonged presentation of antigens could support the notion that GC activity persists for periods of time yet to be defined after vaccination, thus promoting the generation of antibodies with greater diversity and affinity for epitopes of the S protein. Although GC activity in tissues from Sputnik V vaccinated individuals remains to be elucidated, the same mechanisms observed in mRNA vaccines may operate to counteract SARS-CoV-2 variants.

A subset of sera exhibited higher neutralization titers to the Gamma VOC compared to the ancestral variant. This observation suggests that these individuals might have been exposed to this variant during the study period, as it was disseminated in Venezuela during March–August 2021 [16,48]. None of the volunteers reported an infectious event during the clinical trial. These sera showed no reactivity to the N protein [19], which would have been expected in the event of infection, and a modest increase in reactivity to S, but not to RBD Gamma VOC. We suggest that they might have had an asymptomatic or subclinical infection related to the protective effect of the vaccine received, manifested only in the presence of neutralizing antibodies against the infecting variant and a modest increase in reactivity to the Gamma S protein variant. Other authors have found neutralizing antibodies in asymptomatic or mildly affected COVID-19 patients, with no detectable IgG antibodies [49]. Similar results (higher titer against the Gamma variant without apparent previous infection) were obtained by another group that studied the neutralization of Sputnik V-induced antibodies in a cohort of volunteers in Argentina [50]. On the other hand, the fact that the sera from our study exhibited just a modest increase in reactivity toward the S protein and not the RBD region might be due to the fact that other regions of the S protein, like the N-terminal region, might be contributing to inducing neutralizing antibodies against the virus, as suggested by our previous study [19], by the moderate positive correlations found between reactivities and PRNT and previously documented within NTD region, heptad repeats, and the fusion peptide [13,51].

IgA and IgM responses were not determined in this study, which may represent a limitation of this research [52]. However, the reduced number of samples for 180 dpv might also have affected the comparison at this particular point and between 42 dpv. Finally, the inclusion of more viruses from different Omicron lineages in our design could have offered further information on immune escape [53]. Another limitation of this study is that the vaccine studied was designed with the ancestral variant; since then, new Omicron sub-lineages have been used for the design of the vaccine, providing more adequate protection against the circulating lineages. However, as stated before, none of these vaccines, based on new variants, are available in Venezuela.

Taken together, our results suggest that the Sputnik V vaccine induces antibodies that are similarly able to interact with S and neutralizing SARS-CoV-2 variants, with the Omicron VOC being the most resistant to antibody immunity, as previously reported [15,26]. Some volunteers may have been exposed to the Gamma variant during the clinical trial without showing symptoms. This might be associated with the significant beneficial effect of COVID-19 vaccines in protecting, if not completely against, infection, symptomatic infection due to variant emergence, and then against severe disease manifestation, death, and long-term sequelae [54,55]. The antigenic distance between different variants enhances our understanding of how immunized populations may be susceptible to the emergence of new variants, and should continue to be considered in the design of the next generation of vaccines [8,56]. Therefore, continuous monitoring of SARS-CoV-2 evolutionary trajectories should be a priority in the design of new vaccines that include immunogens that promote GC responses to provide broader protection against infections and diseases caused by emerging variants. We provide further evidence on the magnitude, breadth, and persistence of antibodies induced by this adenoviral vaccine in the context of SARS-CoV-2 evolution, thereby providing insights into the design of future vaccines against new potential coronaviruses.

## Figures and Tables

**Table 1:** Number of PUBMED entries per number of countries where the main COVID-19 vaccines were approved.

Vaccine	Manufacturer	Country	PubMed Entries	No. of Countries Vaccine Approved
BNT162b2 (Comirnaty)	Pfizer−BioNTech	Mainz, Germany	14,309	149
ChAdOx1 nCoV-19 (AZD1222)	Astra Zeneca−Oxford University	Cambridge, England	5911	149
Ad26.COV 2-S (Jcovden)	Janssen Biotech, Inc. (Johnson & Johnson)	Beerse, Belgium	4088	113
mRNA-1273 (Spikevax)	Moderna	Cambridge, MA, USA	4824	88
CoronaVac	Sinovac	Beijing, China	1223	56
BBIBP-CorV (Covilo)	Sinopharm	Beijing, China	725	93
NVX-CoV2373 (Nuvaxovid)	Novavax	Gaithersburg, MD, USA	989	40
Gam-COVID-Vac (Sputnik V)	Gamaleya National Center of Epidemiology and Microbiology	Moscow, Russia	563	74

A search query was performed in PubMed using the following words and operators: (COVID-19) OR (SARS-CoV-2) AND (“name of the vaccine”) OR (“name of the manufacturer”) AND (vaccine). The number of countries where the vaccine was approved was assessed as reported by Kudlay and Svistunov, and the COVID-19 vaccine tracker team [17,18].

## Data Availability

The complete genome sequences of the SARS-CoV-2 variants used for neutralization assays have been deposited in the GISAID database accession numbers: ancestral (EPI_ISL_6980947), Gamma (EPI_ISL_2628299), Delta (EPI_ISL_6976265), and Omicron (EPI_ISL_17389567).
